# Hormone variation in *Robinia pseudoacacia* L. (Fabaceae) leaves during gall formation by *Obolodiplosis robiniae* (Haldeman) (Diptera: Cecidomyiidae)

**DOI:** 10.1038/s41598-026-38156-9

**Published:** 2026-03-12

**Authors:** Aleksandra Maria Staszak, Agata Kostro-Ambroziak, Aneta Sienkiewicz, Alicja Piotrowska-Niczyporuk

**Affiliations:** 1 Laboratory of Plant Physiology, Department of Plant Biology and Ecology, Faculty of Biology, University of Bialystok, Ciołkowskiego 1 J, 15-245, Białystok, Poland; 2 Division of Biodiversity and Behavioural Ecology, Department of Genetics and Zoology, Faculty of Biology, University of Bialystok, Ciołkowskiego 1 J, 15-245, Białystok, Poland; 3 Department of Agri-Food Engineering and Environmental Management, Faculty of Civil Engineering and Environmental Sciences, Bialystok University of Technology, Wiejska 45E, Bialystok, 15-351 Poland; 4 Laboratory of Plant Biochemistry, Department of Plant Biology and Ecology, Faculty of Biology, University of Bialystok, Ciołkowskiego 1 J, 15-245, Białystok, Poland

**Keywords:** Insects, Cytokinins, Brassinosteroids, Salicylic acid, Leaf marginal rolling gall, Tree, Biochemistry, Plant sciences, Zoology

## Abstract

Gall-inducing insects modify plant metabolism to convert host tissue cells into new forms of galls. The black locust gall midge *Obolodiplosis robiniae* (Haldeman) forms marginal rolling gall on black locust leaves (*Robinia pseudoacacia* L.). We investigated the hormonal changes in host plants after infection and gall growth to senescence. A wide range of phytohormones was analysed for the first time in gall tissue. Hierarchical cluster analysis demonstrated that young gall (YGall) composition corresponds with non-galled leaflets control (NGLC) and non-galled leaflets of galled leaves (NGLG). A second cluster was formed by mature gall (MGall) and senescent gall (SGall). Through all stages of gall formation the level of hormones increased ABA, GA_3_, auxins (IAA and PAA), different types of brassinosteroids, 7-oxalactone (BL, EBL, and HBL), 6-oxo type (CS, ECS, TY, and CT), and 6-deoxo type (6dTY) as well as different types of cytokinins, such as free bases (*c*Z, *t*Z, DHZ, and iP), *N*-glucosides (*t*Z7G, *t*Z9G, DHZ7G, DHZ9G, and iP7G), *O*-glucosides (*c*ZOG, *c*ZROG, *t*ZOG, *t*ZROG, DHZOG, and DHZOGR), and ribosides (*c*ZR, *t*ZR, iPR, and DHZR), and salicylic acid (SA). There was no reduction in the examined hormones. The senescence galls present the highest fluctuation and we also noticed changes in NGLC and NGLG. We noted the highest levels of HBL and DHZOG and the lowest contents of ABA, auxins, brassinosteroids, cytokinins, GA_3_, and SA. MGall and SGall presented changes in opposite directions. The level of cytokinins in gall tissue was higher than NGLC and NGLG, indicating that infection modulates the levels of these phytohormones in whole compound leaves. DHZOG, *t*ZROG level increased in NGLG variant suggests that they are crucial after infection. Brassinosteroids (HBL) level decreased during YGall formation according to NGLC. Results of wide hormone profiling suggest that hormonal changes occur in sequence.

**Supplementary Information:**

The online version contains supplementary material available at 10.1038/s41598-026-38156-9.

## Introduction

The black locust (*Robinia pseudoacacia* L., Fabaceae) is a non-native European tree species known as one of most invasive species^1^. Furthermore, it is widely used in central and Eastern Europe for wood and fibre production^2,3^. Changes in the occurrence of black locusts in Europe have led to a shift in the range of species that use black locusts as vector trees, such as insects^4,5^. Since 2002, the black locust gall midge (*Obolodiplosis robiniae* (Haldeman, 1847), Diptera: Cecidomyiidae) has been detected outside the United States, e.g., in Japan, Korea, Italy, Asia, and Europe. In Poland, the first observations were made in 2017^6–11^. In an earlier study, we characterised gall formation’s structural and biochemical composition^12^. Herein, we present a broad overview of hormone profile changes during different stages of the gall life cycle. We chose the same stage of gall formation for this analysis. *O. robiniae* induces the formation of marginal rolls on black locust leaflets. The edge of the leaflets curls downwards to create a larval chamber^13^. The larva remains inside the gall and could potentially come out of this chamber. *O. robiniae*, according to environmental factors, may produce various numbers of generations, 2–3 (Japan^6^, 3–4 (Europe^10,14,15^ or 4–6 (China^11^. In one leaf gall, it is possible to find 1–10 larvae^10,16^.

Plant hormones are essential signalling molecules involved in regulating plant growth and proliferation^17^. The gall induction hypothesis predicts three possible options for gall formation. (1) Female insects may produce and leave hormones on the plant tissue surface during oviposition. For example, cytokinins such as *t*ZR and iPR, their free bases, nucleotides, and glucoside forms; and auxins (e.g. IAA) were detected in the salivary glands of *Eurosta solidaginis*^18^ and in the *Pontania* sp. wasp, where accessory glands associated with the ovipositor consist of high levels of cytokinins and auxins^19^. (2) In the second version, ove secretes plant hormones, which can change the organisation of leaflet tissue into the gall form (for more^19–33^. (3) The last hypothesis states that gall formation involves specific proteins and gene expressions responsible for hormone biosynthesis (for additional information, see^34–37^. During our study, we did not work on gall induction processes, but we tried to quantify and understand the role of phytohormones during the different developmental stages of the gall structure. We determined changes in the contents of ABA, GA_3_, SA, auxins (IAA, IBA, IPA, and PAA), different types of BRs (7-oxalactone type BL, EBL, HBL, and NorBL), 6-oxo type (ECS, and CT), and 6-deoxo type (6-deoxytyphasterol]), and different types of cytokinins (free bases *c*Z, *t*Z, DHZ, iP, *o*T, *mT*, *p*T, BA), *N*-glucosides (*t*Z9G, DHZ7G, DHZ9G, and iP7G), *O*-glucosides (*c*ZROG, *t*ZOG, *t*ZROG, DHZOG, and DHZOGR), and ribosides (*c*ZR, *t*ZR, and DHZR) in the development of gall. We aimed to understand the role of phytohormones by providing a comprehensive overview of hormonal variation across different gall developmental stages (young-YGall, maturation-MGall, and senescence-SGall) in non-galled leaflets (NGLC) and non-galled leaflets of galled leaves (NGLG) of *R. pseudoacacia.*

## Results

### Hormonal imbalance during gall formation

During gall formation, we choose three distinct phases as YGall when the marginal rolling gall is formed. It is shown that the margins of leaflets are rolled downwards, MGall when formed gall is swollen and light green, an increase in volume comapred to YGall and SGall when first brown spots are noted (for more of the histological and biochemical description see^12^.

We notice that the level of sort hormones was higher in a specific stage according to comparison to NGLC as NorBL, *t*ZOG, *t*ZOGR, *t*z9G, DHZ, DHZOG, DHZ7G, DHZ9G. Higher level of examined ECS, *c*ZR, *c*ZOGR, IPR7G, *p*T, SA were noticed in all stages comprised to NGLG than to NGLC (Supplementary Table A1).

In YGall we observed that level of IBA, ESC, *c*ZR, *c*ZOGR, IPR7G, *p*T and SA were lower in NGLG than in NGLC. Level of NorBL, *t*ZR, *t*ZOG, *t*ZOGR, *t*z9G, DHZ, DHZOG, DHZ7G, DHZ9G were lower in NGLC than other stages.

In MGall and SGall the level of mentioned hormons enrich their level but profile of changes has the same trend. The level of hormones is higher in SGall than in MGall. When we take into account cytokines: *t*ZOGR, DHZ7G, IP we observed that their level in MGall and SGall rised according to YGall when we compare results with level in NGLG.

The hormonal profile presented as peaks generated by liquid chromatography ‒ mass spectrometry (LC‒MS) analyses were sown in Supplementary files as A2.

**Fig. 1:**
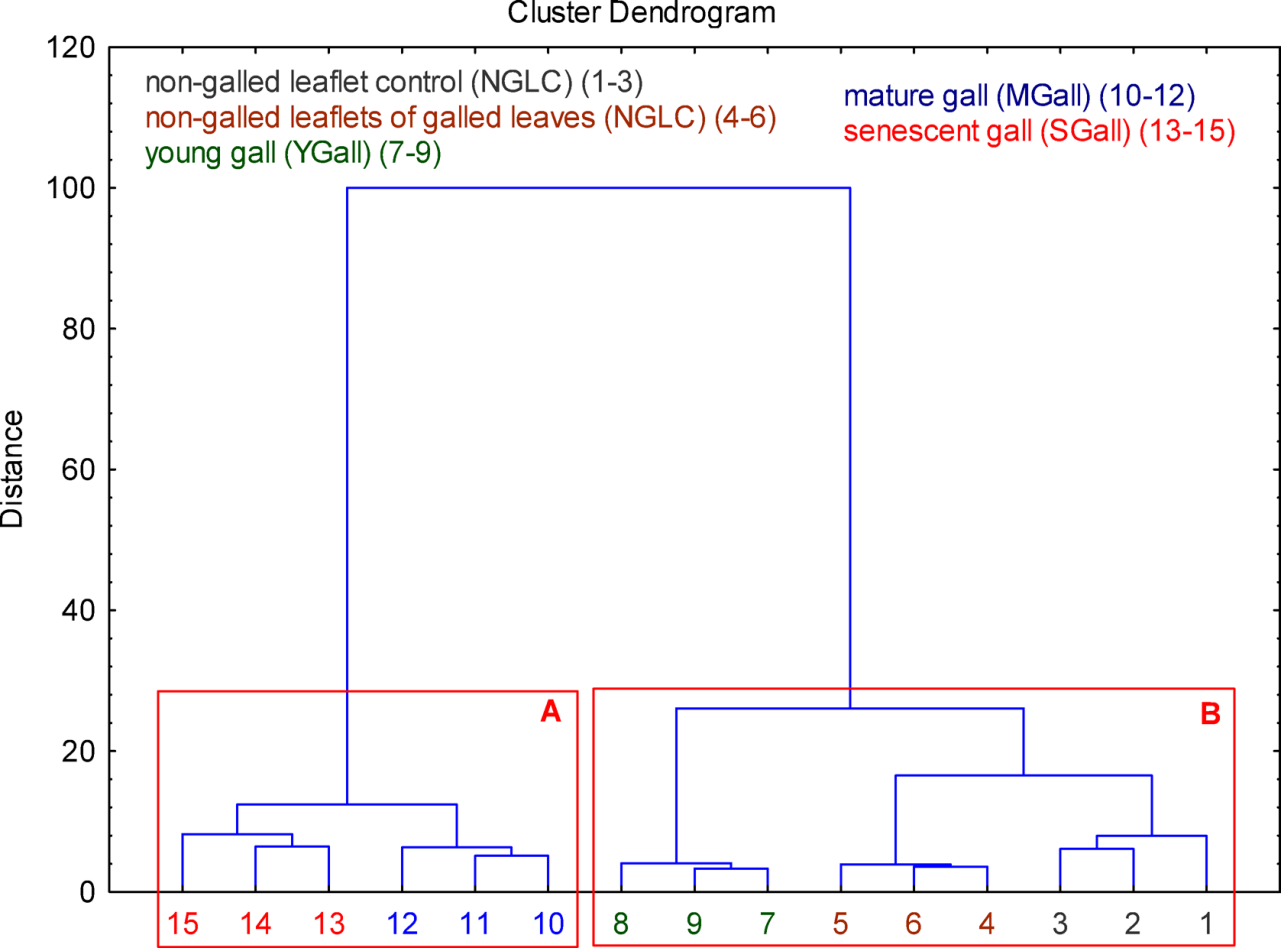
Dendrogram of the hierarchical cluster analysis (HCA) of the tested samples. Final partition: Cluster A: mature galls (MGall), senescent galls (SGall); Cluster B: non-galled leaflet control (NGLC), non-galled leaflets of galled leaves (NGLG), young galls (YGall). Numbers 1–3 for NGLC, 4–6 for NGLG, 7–9 for YGall, 10–12 for MGall, and 13–15 for SGall.

According to hierarchical cluster analysis (HCA), we grouped the tested samples based on plant hormone content into two clusters (A–B) (Fig. 1). In Cluster A, where MGall and SGall were combined, the contents of ABA, auxins, brassinosteroids, cytokinins, GA_3_, and SA were more significant than the average in a given group; however, the contents of HBL and DHZOG were lower in this cluster. Cluster B included NGLC, NGLG, and YGall, which, despite having the highest HBL and DHZOG contents, were characterised by the lowest ABA, auxins, brassinosteroids, cytokinins, GA_3_, and SA contents.

**Fig. 2:**
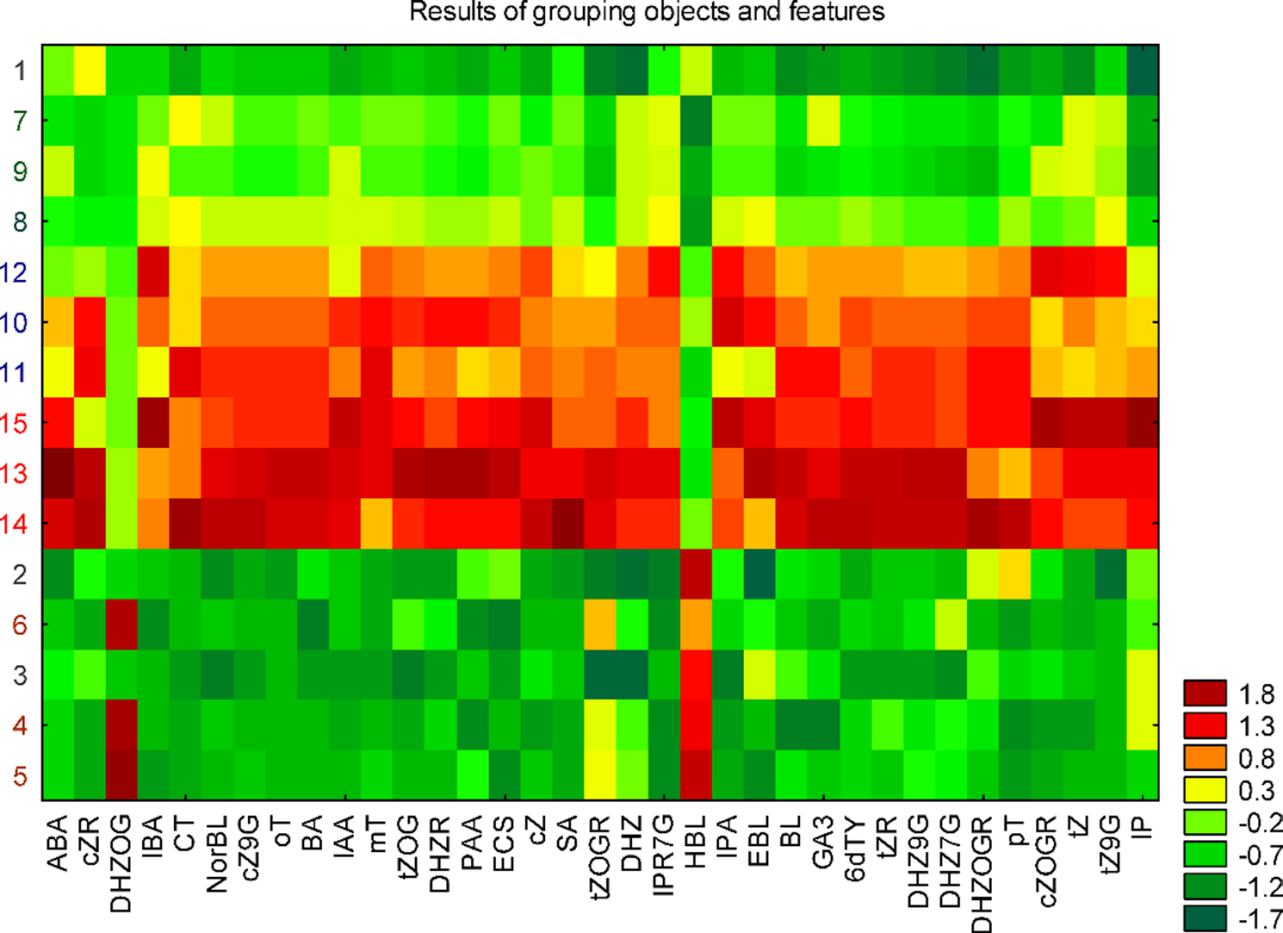
Graphical result of the simultaneous grouping of objects (tested samples; the numbers correspond to the samples from the dendrogram) and features (the content of plant hormones). Numbers 1–3 for NGLC, 4–6 for NGLG, 7–9 for YGall, 10–12 for MGall, and 13–15 for SGall.

Using grouping object and feature analysis, we confirmed that our results could be divided into two groups of objects with an extreme lack of similarity (Fig. 2). The central part of the map features an area where MGall and SGall were grouped (red). Conversely, in the upper and lower parts of the map, the samples with the lowest contents of the plant hormones NGLC, NGLG and YGall were grouped (green). This analysis also showed that DHZOG and HBL were displaced from the central to the lower part of the map.

**Fig. 3:**
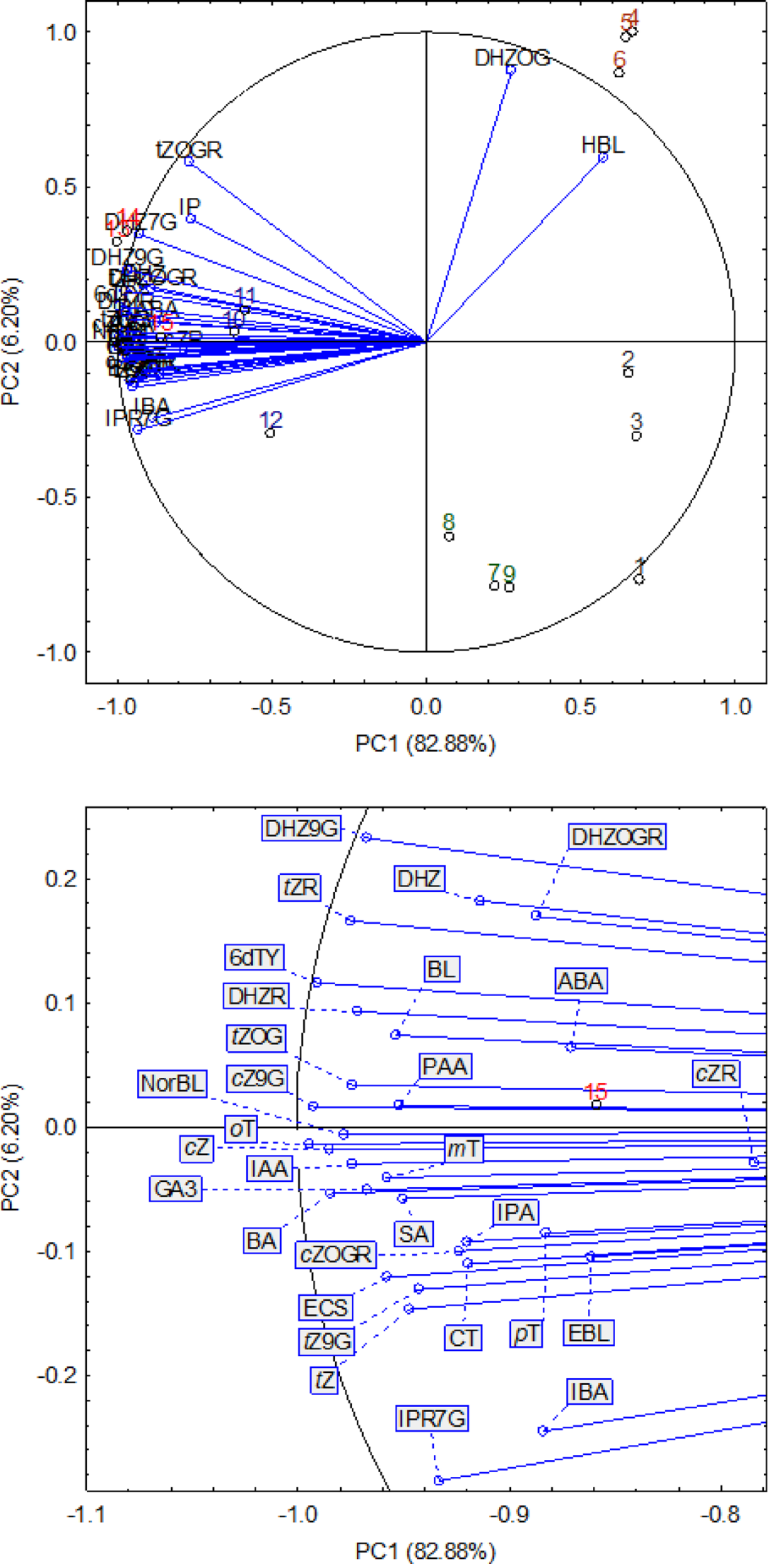
Biplot of plant hormone content for each repetition (*n* = 3) in five types of samples, showing the first two principal components (PC1 and PC2) of the PCA model that together explain 89.1% of the total variance (82.9% and 6.2% for PC1 and PC2, respectively). The blue biplot vectors indicate the strength and direction of factor loading for all the analysed variables. The numbers correspond to the samples from the dendrogram. Numbers 1–3 for NGLC, 4–6 for NGLG, 7–9 for YGall, 10–12 for MGall, and 13–15 for SGall.

Further principal components analysis (PCA) allows the clustering of analysed samples, preserving a high level of explained variance. In this analysis, the number of variables was reduced to two principal components (PC1 and PC2), suggesting that the 35 plant hormones in the dataset are highly correlated and can be reduced. All variables (except DHZOG and HBL) had high negative loadings (from − 0.9944 to −0.7603) with the first component. In turn, DHZOG and HBL had high positive loadings (0.8759 and 0.5960, respectively), similar to the second component. The values of factor loadings for almost all variables were very close, and on one graph, these points overlapped (Fig. 3).

**Fig. 4:**
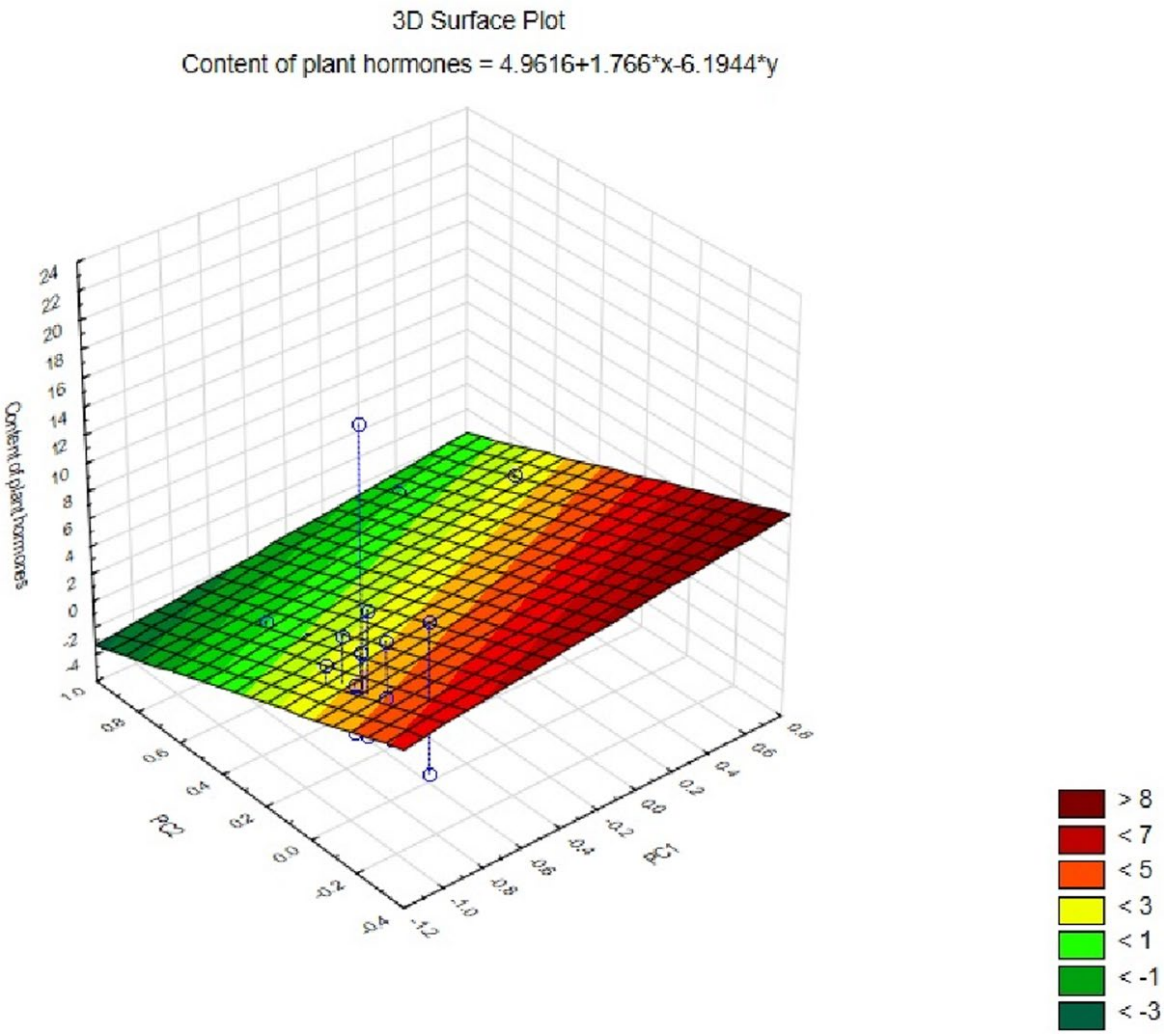
A 3D-surface plot showing the relationships between the contents of plant hormones, PC1 and PC2.

After comparing the positions of the samples on the graph with the forms of the components and factor loadings, it was found that mature galls and senescence galls (negative values of coordinates for the first axis) contained (considering all factor loadings except for DHZOG and HBL with the first axis) by a larger content of plant hormones. An NGLG (positive values of coordinates for the second axis) was used (considering factor loadings with the second axis) because of the more significant content of DHZOG and HBL. The NGLC and YGall samples, depending on the position on the graph, had significantly lower contents of plant hormones (Fig. 3). The relationships between the contents of phytohormones and two principal components (PC1 and PC2) are presented in a three-dimensional surface plot (Fig. 4). The regression surface illustrates the overall trends in the dataset as well as the spatial distribution of the measured points. The analysis shows that PC1 is positively associated with phytohormone content, with higher PC1 values corresponding to increased predicted hormone levels. In contrast, PC2 exhibits a strong negative effect, whereby increasing PC2 values leads to a pronounced decline in the estimated hormone content. This pattern is clearly reflected in the downward slope of the regression surface along the PC2 axis. The highest hormone levels, represented by warmer colours, occur in samples with low PC1 values (approximately − 0.9) and PC2 values close to zero or slightly negative. Conversely, the lowest concentrations depicted in green shades are observed primarily in samples with higher PC2 values, and moderately elevated PC1 values. The close alignment between the experimental data points and the regression surface indicates that the linear model effectively captures the principal relationships present within the dataset.

## Discussion

### General background in gall formation on *R. pseudoacacia*

The black locust gall midge *Obolodiplosis robiniae* forms rare marginal rolling gall on compound black locust leaves (*Robinia pseudoacacia*)^13^. The gall-inducing insect must transcend the plant’s defence lines to induce the gall. In the plant that produces compouned leaves, the reaction of specific leaflets for gall formation may be distinct.

Reprogramming of normal cycle of plant leaves tissue according to gall induction is possible according to changes caused by mother insects or/and egg or and feeding larvae. We identified changes in hormonal composition during three phase of marginal rolling gall on *R. pseudoacacia*. During hormonal analysis, we chose two kinds of leaflets for analysis: NGLC and NGLG. In previous work^12^, we identified that in NGLC, the levels of hydrogen peroxide and phenol, as well as non-protein -SH, are higher than in NGLG. In this variant more protein with -SH grouped were noted, also catalase activity was higher as well tannin levels. We don’t detect statistical important changes in leaflets with one or more gall formed on the leaflets. The hierarchical cluster analysis and grouped analysis shown that NGLC and NGLG shared more commons with YGall than with other two stages.

Further analysis in Staszak et al.^12^ of histology and biochemistry on gall leaflets and gall isolated were done in stage comparable with MGall in this work. In gall tissue level of hydrogen peroxide was the lower amount in all examined variant as well catalase activity, also phenol and tannins levels was lower than in other variants. It gives a clue that inside the gall the defence system is diminished. The composition of biochemical changes showed that NGLC showed more hydrogen peroxide and phenols, levels according to NGLG, where on some other leaflets, invasive studies were done. This suggests gall insects during invasion modulate the defence systems of the host plant. Observation of multiple gall formation on infected leaves may be connected with this modification.

### Hormonal profiles changes

Auxins take part in plant growth and development^38^. NGLC presented higher levels of auxins, such as IBA, PAA, than those from NGLG. During gall formation, accumulations of auxins such as IBA, IPA, and PAA were noted. We also observed fluctuations in the IBA concentration. Its level was slightly lower in the NGLG than in NGLC (Supplementary Table A1) but was higher in the gall tissue. After gall induction, the level of IBA dropped, and an intensive process of growth and development of the margin roll gall was started. Like in our study, the content of IAA was strongly induced in herbivore-attacked *Nicotiana attenuata* plants^39^. These results showed that IAA is required for the herbivore-specific and jasmonic acid-dependent accumulation of plant secondary metabolites such as anthocyanins and phenolamides in stems^39^. Like in *R. pseudoacacia*, the level of the auxin IAA was elevated in plants attacked by gall-feeding insects, indicating that larvae may trigger of IAA production in developing galls^21,40,41^. In YGall intensive cell proliferation occurs forming the marginal roll gall morphotype[for more details see^12^. However, auxin may also intervene in resources allocation as shown in research on fig syconial development under parallel formation of fig wasp gall and seeds^41^.

We observed that the levels of free bases and ribosides of cytokinins were greater during gall formation than during the other stages, when the levels of *N*-glucosides and *O*-glucosides increased. Generally, the accumulation of cytokinins in gall tissue was more significant than that in NGLG. This means that insect attack modulates the level of cytokinins in whole black locust leaves. Our research indicated that the amount of *t*Z was most stimulated during gall formation in *R. pseudoacacia* in response to *O. robiniae* attack. Several lines of evidence also support the critical role of cytokinins in activating plant defence against herbivore attack. For example, cytokinins such as *t*Z and iP were immunolocalised in the *E. solidaginis* salivary gland. They are located in the cytoplasm of the cell and cell membrane respectively, this species produces galls on *Solidago altisima*^18^. During an experiment on the larch *Adelges laricis* and in the gall tissues of *Picea koraiensis*, the levels of *t*Z and iP were low, in contrast to the level of BA, which regulates this kind of gall formation^42^. In case of gall induction by many other organisms, the tRNA-ipt pathway is activated^43–45^, which is deeply involved in the production of bioactive cytokinins such as *t*Z and iP. In particular, in plant-feeding organisms (not only those producing gall tissue), the tRNA-ipt pathway is probably more efficient at producing cytokinins than in the plant kingdom^33^. Such a pattern would undermine the notion that the tRNA-ipt pathway is unproductive and yields only *cis*-zeatin-type phytohormones, as claimed in the literature on cytokinin biosynthesis in plants^46–49^. We concur with the suggestion that the tRNA-ipt pathway functions differently and is considerably more efficient in insects than in plants to produce cytokinins^33^.

The *c*ZR level is also modulated *via* infection by *O. robiniae* in *R. pseudoacacia.* The amount of this cytokinin was reduced in NGLG and YGall after insect attack, whereas its level increased during gall growth and ageing. In sawflies (*Pontania* sp.) that produce galls on *Salix japonica*, high levels of *t*ZR were detected. The transcript levels of genes involved in auxin and cytokinin biosynthesis and signalling were greater in galls than in uninfected leaf tissues^19^.

Among the cytokinins, dihydrozeatin-*O*-glucoside (DHZOG) is a biologically inactive and stored form of DHZ. The level of DHZOG was the highest in NGLG, whereas in gall tissue, its level decreased, leading to an increase in its free base DHZ. Most likely, the hydrolysis of conjugated cytokinins to their free forms occurs during gall formation. DHZ is crucial for cell division, leaf expansion, and delaying leaf aging^51,52^. Increasing level of DHZOG may be a sign of attractors for further gall forming on NGLG leaflets. The opposite results were noted in maize corn (*Ustilago maydis*) after fungal infection, where the level of DHZOG increased after infection^53^. During the development of early roots and hypocotyl growth in *Theobroma cacao*, the DHZOG levels are stable, and the only one recorded form of cytokinin is DHZ^54^. In transgenic tobacco (*Nicotiana tabacum* cv. Xanthi nc) plants may accumulate high levels of cytokinins after insect wounding. The authors suggest that plant resistance to insects depends on cytokinins levels and leaf ontogeny^55^. For example, high levels of cytokinins are observed in young developing leaves, which exhibit intense cell division, whereas reduced cytokinins content is associated with leaf senescence^56,57^. Thus, cytokinins may be involved in herbivore-induced defences mechanisms, tissue-specific responses, and gall formation in *R. pseudoacacia* leaves where intensive cell proliferation occurs, as shown in our previous studies^12^. We notice fluctuation in level of cytokines as DHZ, *t*ZOG, *t*ZOGR, *t*z9G, DHZ, DHZOG, DHZ7G, DHZ9G examined stages were higher in NGLG. On the other hand level of *c*ZR, *c*ZOGR, IPR7G, *p*T In NGLC rise in YGall and other stages. In MGall and SGall the level of mentioned hormones enrich their level but profile of changes has the same trend. The level of hormones is higher in SGall than in MGall.

Brassinosteroids may act as plant hormones, inducing abiotic stress tolerance and improving plant growth and development^58^. They also participate in plant acclimation to unfavourable environmental conditions and resistance to pathogens^59^.

The literature contains only a few reports on the level of brassinosteroids during gall formation earlier reports shown presence of castasterone that was isolated from gall tissue in chestnut^60^. Infection of *R. pseudoacacia* by *O. robiniae* led to decreased CT, BL, and ECS levels in the NGLG. During biochemical analysis we observed lower level of phenols in this kinde of tissue^12^.

The HBL levels in NGLC and NGLG were high, whereas in YGall, the HBL content decreased to approximately half of the control level and remained low in MGall and SGall. This suggests that it is directly correlated with insect egg deposition and induction of gall development. The levels of other BRs (EBL, NorBL, and 6-dTY) increased, especially those of MGall and SGall. At this stage, cell maturation is observed, as well as induction of sclerenchyma in the cell wall occurs^12^.

Brassinosteroids take part in plant adaptation to abiotic stress. Their ability to detoxify heavy metals (more^59^ by reducing their uptake through the alteration of cell permeability and the reduction of damage by activating defensive enzymes has been confirmed in many plants^61^. HBL levels increase in plant tissue under heavy metal stress, such as aluminium (mung bean shoots and roots, chlorophyll content)^62^, nickel (indian mustard, seed germination)^63^, and chromium (*Raphanus sativus* seeds and seedlings)^64^. HBL not only reduces the influence of metal stress^65–68^ but also participates in inducing defence mechanisms and photosynthesis processes in response to abiotic stress^69,70^. Many experiments involving the external application of HBL to leaves or EBL to soaked seeds assumed that the photosynthetic rate was enhanced by increasing the chlorophyll content^71^. However, the role of brassinosteroids in interactions between plants and insects has not been fully elucidated. Some published data indicated that brassinosteroids play a negative role in the rice’s (*Oryza sativa*) defence against the brown planthopper (*Nilaparvata lugens*) because insect attack suppresses the brassinosteroid pathway. In contrast, the salicylic acid (SA) pathway is activated^72^. The concentrations of brassinosteroids significantly decreased in response to the presence of insects. On the other hand, brassinosteroid-overproducing rice plants exhibit greater tolerance, as indicated by an increased seedling mortality rate after infestation with *N. lugens*, and *the* nymph survival rate is significantly lower in comparison with that of the wild type^72^. However, similar to our findings, brassinosteroids have also been found to act as positive regulators of defence against the chewing herbivore *Manduca sexta* and the cell-content feeder *T. tabaci*, which employ very different feeding methods for phloem-feeding *N. lugens*^73,74^.

### Other hormonal changes

The levels of ABA, GA_3_, and SA are lower in the NGLG leaflets of *R. pseudoacacia* than those in the GCs leaflets. However, the gall tissue was characterised by a high accumulation of these plant hormones after the *O. robiniae* attack.

In contrast to our results, in the maize orange leafhopper *Cicadulina bipunctata* the level of gibberellins from GA_1_ to GA_4_ decreased during gall formation^75^. However, other studies suggest that gibberellins play a key role in regulating gall formation and insect-induced defensive responses^76^. For example, the concentrations of gibberellins (GA_1_, GA_3_, and GA_4_) increased in galls present Korean spruce (*P. koraiensis*) induced by the larch adelgid (*A. laricis laricis)*, which is a specialist insect parasite of this plant species^42^. Thus, the hyperplasia and hypertrophy of the gall tissues observed in *R. pseudoacacia* leaflets might be stimulated by GA_3_ synthesised in plant tissue in response to *O. robiniae* attack^12^. On the other hand, studies on rice (*Oryza sativa*) plants infested with brown planthoppers (*N. lugens*) characterised growth suppression correlated with decreased GA levels. Herbivores may activate the catabolism of these plant hormones in leaf tissue, inducing the conversion of bioactive GAs to their inactive forms^77^. Similarly, studies on plants attacked by larvae have shown changes in GA_3_ accumulation and signalling, suggesting a key role for GA in herbivore-induced defence responses. For example, DELLA proteins are negative transcriptional regulators of GA-induced gene expression and are involved in plant responses to diverse developmental and environmental factors^78^. Moreover, the accumulation of GAs during gall formation affects the signalling of other phytohormones (jasmonic acid, JA) through competitive binding of DELLAs to JAZ proteins, thereby preventing JAZ–MYC2 interactions and promoting MYC2-induced transcriptional responses, leading to plant tolerance to herbivore attack^79^.

The level of ABA in the SGall was two times greater than in the NGLC treatment. The modulation of ABA level by gall formation insects were described earlier in *C. bipunctata*, the level of ABA increased during gall formation in newly extended leaves during the feeding of leafhoppers and decreased after the removal them from the leaves^75^. Elevated levels of ABA were also observed in *Arabidopsis thaliana* infected with the specialist small cabbage white (*Pieris rapae*) and the generalist Egyptian cotton worm (*Spodoptera littoralis*). This study revealed the key role played by ABA in defence reactions against insects in plants and identified some components essential for plant resistance to herbivory^80^. Published data suggest that ABA may be present at high concentrations in gall-inducing species of insects. ABA was found in the salivary glands of the larvae of the gall-inducing *E. solidaginis*. Therefore, the authors suggest that insects may synthesise and secrete ABA to manipulate their host plant anatomy and physiological processes, such as source-sink mechanisms of nutrient allocation^81^.

The increase in SA content during gall formation in *R. pseudoacacia* leaflets in response to the *O. robiniae* insect confirms that this is an important plant hormone that mediates plant defence reactions against biotrophic and hemibiotrophic pathogens and some insects^82^. This phytohormone triggers related defence responses and increases the field’s disease resistance and plant fitness^83^. Similar to our findings on *R. pseudoacacia* infected by *O. robiniae*, increased levels of SA were observed in *A. thaliana* plants infested with the *Eurydema oleracea*^84^ herbivore and in tomato (*Solanum lycopersicum*) plants attacked by Colorado potato beetle (*Leptinotarsa decemlineata*) larvae^85^. Moreover, herbivore attack triggers signalling pathways and alters gene expression in plant defence responses to biotic stress^84^. The *Jacobaea aquatica* leaf tissue treated with SA was characterised by reduced damage to feeding by the piercing-sucking herbivore (*Frankliniella occidentalis*)^86^.

## Conclusion

These results are the first reports of phytohormonal changes caused by *O. robiniae* during the developmental stages of marginal roll galls on black locust compound leaves. The level of cytokinins in gall tissue was higher than NGLC and NGLG, indicating that infection modulates the levels of these phytohormones in whole compound leaves. DHZOG, *t*ZROG level increased in NGLG variant suggests that they are crucial after infection. Brassinosteroids (HBL) level decreased during YGall formation according to NGLC what give an open question for further analysis on role of this kind of plant regulator during gall formation. Similarly, the levels of ABA, GA_3_, and SA increased during gall formation, resulting in enhanced plant adaptation and tolerance to insect attack. Results of wide hormone profiling suggest that hormonal changes occur in sequence. Young form of gall tissue share more comparable with results of control treatments (NGLG, NGLC) than with mature (MGall) and senescence (SGall) phase. Further analysis on specific role of hormones with usage an inhibitors may give new light on specific transformations in each stage of gall development.

## Materials and methods

### Plant material

The leaflets of *R. pseudoacacia* trees were collected from compound leaves from May to July 2020 and 2021 in Białystok (north-eastern Poland, at the University of Bialystok campus). The Voucher specimen is kept in the database of Vascular plants of north-estern Poland no. UWBB001139. The *R. pseudoacacia* L. is common species in Poland, and the collection of black locust is not subject to any legal restrictions. The first author undertook the formal identification of the plant material used in this study. During the experiment, we tested five groups of plant tissue: NGLC - Non-galled leaflet control, NGLG - Non-galled leaflets of galled leaves, YGall - Young gall, MGall - Mature gall, SGall - Senescent gall. The stages are comparable with previous biochemical and histological analyses on morphology^12^.

A total of 200 mg of plant material was weighed per sample, frozen in liquid nitrogen, and subsequently stored until the hormone profiles were analysed. In variants of YGall, MGall, SGall we only tested the gall part.

### Hormone profiling

#### Chemicals

In the present study, the relationships between gall (young-YGall, mature-MGall, and senescence-SGall) induced by *O. robiniae* and different hormone levels, such as ABA, GA_3_, SA, auxins (IAA, IBA, IPA, PAA), BRs (BL, EBL, HBL, NorBL, ECS, CT, and 6dTY), and cytokinins (*c*Z, *t*Z, DHZ, iP, *o*T, *m*T, *p*T, *t*Z9G, DHZ7G, DHZ9G, iP7G, *c*ZROG, *t*ZOG, *t*ZROG, DHZOG, DHZOGR, *c*ZR, *t*ZR, DHZR, and BA), were studied in detail in *R. pseudoacacia* plants.

All the chemicals used in the phytohormone extraction were purchased from Merck KGaA (Darmstadt, Germany). The solvents used for liquid chromatography ‒ mass spectrometry (LC‒MS) analyses were of high-performance grade. Phytohormone standards, i.e., abscisic acid (ABA), indole-3-acetic acid (IAA), indole-3-butyric acid (IBA), indole-3-pyruvic acid (IPA), phenylacetic acid (PAA), brassinolide (BL), 28-homobrassinolide (HBL), 24-epibrassinolide (epiBL), 28-norbrassinolide (norBL), 24-epicastasterone (epiCS), cathasterone (CT), 6-deoxytyphasterol (6dTY), *cis*-zeatin (cZ), *cis*-zeatin-riboside (cZR), *cis*-zeatin-9-glucoside (cZ9G), *cis*-zeatin-*O*-glucoside riboside (cZOGR), *trans*-zeatin (tZ), *trans*-zeatin-riboside (tZR), *trans*-zeatin-*O*-glucoside (*t*ZOG), *trans*-zeatin-*O*-glucoside riboside (tZOGR), *trans*-zeatin-9-glucoside (tZ9G), *trans*-zeatin-9-glucoside-*O*-glucoside (tZ9GOG), dihydrozeatin (DHZ), dihydrozeatin riboside (DHZR), dihydrozeatin-*O*-glucoside (DHZOG), dihydrozeatin-*O*-glucoside riboside (DHZOGR), dihydrozeatin-7-glucoside (DHZ7G), dihydrozeatin-9-glucoside (DHZ9G), *N*^6^-isopentenyladenine (IP), *N*^6^-isopentenyladenosine-7-glucoside (IPR7G), *o*-topolin (oT), *m*-topolin (mT), *p*-topolin (pT), *N*^*6*^-benzyladenine (BA), gibberellic acid (GA_3_), and salicylic acid (SA) were purchased from OlChemIm (Olomouc, Czech Republic).

#### Phytohormone extraction

For the phytohormone measurement, the method described by Šimura et al.^87^ was used. The leaves were weighed, and 200 mg of biomass was placed into 2 mL Eppendorf tubes. One millilitre of 50% acetonitrile (ACN) was added, and the plant material was homogenised in liquid nitrogen and then in a bead mill homogeniser (3 cycles, 3 min, speed 3.10 m s^− 1^; OMNI International, a PerkinElmer company, Kennesaw, GA, USA) using two 3 mm tungsten balls. Then, the samples were homogenised using an ultrasound processor VCX 130 (power 130 W, frequency 20 kHz, 5 min) equipped with a titanium probe (Sonics & Materials, Inc., Newtown, CT, USA) and mixed in a laboratory shaker (90 rpm, dark, 5 °C, 30 min; LC-350, Pol-Eko-Aparatura, Poland). The samples were centrifuged (9000×g, 5 min; MPW-55, Med. Instruments, Gliwice, Poland) and collected in a glass tube. For the quantification of phytohormones, stable isotope-labelled standards of [^2^H_6_] (+)-*cis*, *trans*-ABA (50 ng), [^2^H_5_] IAA (30 ng), [^2^H_6_] IP (50 ng), [^2^H_5_] *t*Z (30 ng), [^2^H_5_] *t*ZOG (30 ng), [^2^H_3_] DHZR (30 ng), [^2^H_2_] GA_3_ (30 ng), [^2^H_3_] BL (20 ng) and [^2^H_3_] CS (20 ng) were added to the samples as internal standards. The prepared extracts were purged using a Waters SPE Oasis^®^ HLB cartridge (Waters Corporation, Milford, MA, USA), previously activated and equilibrated using 1 mL of 100% methanol (MeOH), 1 mL of H_2_O, and 1 mL of 50% (*v/v*) ACN. Then, the extracts were loaded and collected in Eppendorf tubes and eluted with 1 mL of 30% (*v/v*) ACN. The samples were evaporated to dryness by a centrifugal vacuum concentrator (Labconco CentriVap micro IR; Labconco Corp. Kansas City, MO, USA), dissolved in 50 µL of 30% (*v/v*) ACN, and transferred to insert vials.

#### LC-MS analysis of phytohormones

The targeted compounds were analysed using an LC-MS 8050 system consisting of a pump, degasser, autosampler, column oven, and mass spectrometer with a triple quadrupole (Shimadzu Corporation, Kyoto, Japan). Ten microlitres of each sample were injected into a Waters XSelect C_18_ column (250 mm × 3.0 mm, five µm) (Waters Corporation, Milford, MA, USA) and heated to 50 °C. Mobile phase A was 0.01% (*v/v*) formic acid (FA) in ACN, and phase B was 0.01% (*v/v*) FA in H_2_O; the flow rate was 0.5 mL min^− 1^. Separation of the above hormones was performed in ESI-positive mode with the following gradient: 0–8 min, flow rate increased linearly from 5 to 30% A; 8–25 min, 80% A; 25–28 min, 100% A; and 28–30 min, 5% A. The mobile LC phase consisted of binary gradients of ACN with 0.01% (*v/v*) formic acid (FA) (A) and 0.01% (*v/v*) aqueous FA (B), flowing at 0.5 mL min^− 1^, which depended on the ESI mode, as described below. The analytical data were analysed using Shimadzu Browser Workstation Software for LC-MS (Shimadzu Corporation, Kyoto, Japan).

#### Statistical analysis

The data were analysed using Statistica software (StatSoft, Poland). The statistical analysis was performed using a one-way ANOVA and Duncan post hoc test. The results are also shown as percentages of the general (GC) or internal (IC) control. The level of significance in all statistical tests was *p* ≤ 0.05. Hierarchical cluster analysis (HCA) was applied to construct a dendrogram that grouped the data into a tree of clusters based on the distances between all pairs of objects. A dendrogram of the obtained clusters was created with Euclidean distance, while the agglomerative criterion was set to the Wardʹs method. Next, principal component analysis (PCA) was performed to construct a model of the relationships between variables. The first fourteen factors were preserved in a biplot for further study. The final biplot was created using two principal components (PC1 and PC2), which together explained 89.1% of the total variance. Statistical analyses were performed with Statistica 13.3 software (TIBCO Software, Inc., Palo Alto, CA, USA).

## Supplementary Information

Below is the link to the electronic supplementary material.

Supplementary Material 1

Supplementary Material 2

## Data Availability

All data are included in the manuscript, and supplementary files and any other additional information maybe provided upon request.

## Abbreviations

ABA: abscisic acid
BA: N^6^-benzyladenine
BL: brassinolide
CT: cathasterone
*c*Z: cis-zeatin
*c*Z9G: cis-zeatin-9-glucoside
*c*ZOGR: cis-zeatin-O-glucoside riboside
*c*ZR: cis-zeatin-riboside
6dTY: 6-deoxytyphasterol
DHZ: dihydrozeatin
DHZ7G: dihydrozeatin-7-glucoside
DHZ9G: dihydrozeatin-9-glucoside
DHZOG: dihydrozeatin-O-glucoside
DHZOGR: dihydrozeatin-O-glucoside riboside
DHZR: dihydrozeatin riboside
EBL: 24-epibrassinolide
ECS: 24-epicastasterone
GA_3_: gibberellin
HBL: 28-homobrassinolide
IAA: indole-3-acetic acid
IBA: indole-3-butyric acid
IP: N^6^-isopentenyladenine
IPA: indole-3-propionic acid
IPR7G: N^6^-isopentenyladenine-7-glucoside
*m*T: meta-topolin
NorBL: norbrassinolide
*o*T: orto-topolin
PAA: phenylacetic acid
*p*T: para-topolin
SA: salicylic acid
*t*Z: trans-zeatin
*t*Z9G: trans-zeatin-9-glucoside
*t*ZOG: trans-zeatin-O-glucoside
*t*ZOGR: trans-zeatin-O-glucoside riboside
*t*ZR: trans-zeatin-riboside
