# Supplementary material for: Hormone variation in Robinia pseudoacacia L. (Fabaceae) leaves during gall formation by Obolodiplosis robiniae (Haldeman) (Diptera: Cecidomyiidae)
Source: Sci Rep. 2026 Mar 12;16:8815. doi: 10.1038/s41598-026-38156-9 (PMC12982751; doi:10.1038/s41598-026-38156-9)
Supplement: Supplementary file 1 — Supplementary Material 1 [file 41598_2026_38156_MOESM1_ESM.docx]

Table A1 Phytohormone contents in non-galled leaflet control (NGLC), non-galled leaflets of galled leaves (NGLG), young galls (YGall), mature galls (MGall), and senescent galls (SGall).

| Phytohormone content  (ng/g biomass) | NGLC | | | NGLG | | | YGall | | | MGall | | | SGall | | |
| --- | --- | --- | --- | --- | --- | --- | --- | --- | --- | --- | --- | --- | --- | --- | --- |
| ABA | 3.989 | 2.063 | 3.450 | 3.140 | 3.126 | 2.915 | 3.348 | 3.743 | 4.457 | 5.608 | 4.976 | 4.172 | 9.366 | 7.365 | 6.925 |
| IAA | 15.613 | 16.741 | 15.037 | 15.536 | 16.005 | 16.706 | 19.953 | 22.008 | 22.155 | 28.329 | 26.472 | 22.789 | 30.561 | 30.443 | 31.450 |
| IBA | 7.443 | 7.116 | 6.835 | 6.929 | 5.990 | 5.776 | 9.512 | 10.491 | 11.131 | 13.458 | 11.003 | 15.474 | 12.611 | 12.983 | 17.377 |
| IPA | 5.461 | 6.628 | 4.583 | 5.084 | 5.359 | 5.873 | 6.979 | 7.698 | 6.700 | 10.608 | 8.008 | 9.916 | 9.306 | 9.450 | 11.136 |
| PAA | 2.261 | 2.992 | 2.479 | 2.105 | 2.884 | 2.095 | 2.890 | 3.187 | 2.774 | 4.392 | 3.768 | 3.921 | 5.095 | 4.446 | 4.403 |
| BL | 3.986 | 5.056 | 5.617 | 3.711 | 5.113 | 4.746 | 5.094 | 5.814 | 4.881 | 7.742 | 8.314 | 7.224 | 8.981 | 8.811 | 8.113 |
| HBL | 2.564 | 3.794 | 3.457 | 3.562 | 3.747 | 3.061 | 1.653 | 1.823 | 1.867 | 2.512 | 2.070 | 2.398 | 2.143 | 2.443 | 2.251 |
| EBL | 2.514 | 1.426 | 3.552 | 2.341 | 2.005 | 3.001 | 3.213 | 3.869 | 3.084 | 4.884 | 3.533 | 4.565 | 5.665 | 4.169 | 5.126 |
| NorBL | 2.083 | 1.498 | 1.313 | 1.940 | 1.882 | 1.943 | 2.986 | 2.937 | 2.556 | 4.025 | 4.323 | 3.783 | 4.669 | 5.102 | 4.248 |
| ECS | 2.894 | 3.744 | 2.428 | 2.695 | 2.321 | 2.052 | 3.699 | 4.080 | 3.551 | 5.623 | 4.835 | 5.256 | 6.522 | 5.705 | 5.902 |
| CT | 1.692 | 1.892 | 1.562 | 1.644 | 1.663 | 1.784 | 4.008 | 3.976 | 2.849 | 4.093 | 5.685 | 4.217 | 4.748 | 6.709 | 4.736 |
| 6dTY | 2.627 | 2.662 | 2.451 | 2.932 | 2.995 | 2.971 | 3.358 | 3.703 | 3.223 | 5.103 | 5.077 | 4.770 | 5.920 | 5.990 | 5.357 |
| cZ | 1.747 | 1.704 | 2.136 | 1.627 | 1.876 | 1.805 | 2.233 | 2.463 | 2.435 | 3.394 | 3.522 | 3.604 | 3.937 | 4.156 | 4.047 |
| cZR | 0.199 | 0.126 | 0.139 | 0.076 | 0.073 | 0.079 | 0.105 | 0.116 | 0.101 | 0.265 | 0.267 | 0.149 | 0.307 | 0.316 | 0.167 |
| cZ9G | 1.313 | 1.142 | 1.111 | 1.222 | 1.284 | 1.255 | 1.678 | 1.851 | 1.611 | 2.550 | 2.647 | 2.384 | 2.959 | 3.123 | 2.677 |
| cZOGR | 0.758 | 0.932 | 0.945 | 0.706 | 0.771 | 0.798 | 0.969 | 1.069 | 1.301 | 1.473 | 1.528 | 1.926 | 1.708 | 1.803 | 2.162 |
| tZ | 1.462 | 1.772 | 2.039 | 1.559 | 1.993 | 1.723 | 3.869 | 3.061 | 3.794 | 4.880 | 4.377 | 5.615 | 5.661 | 5.165 | 6.306 |
| tZR | 0.126 | 0.141 | 0.124 | 0.176 | 0.142 | 0.145 | 0.166 | 0.183 | 0.159 | 0.252 | 0.262 | 0.236 | 0.298 | 0.294 | 0.262 |
| tZOG | 0.558 | 0.484 | 0.426 | 0.521 | 0.544 | 0.703 | 0.736 | 0.812 | 0.707 | 1.120 | 1.008 | 1.046 | 1.321 | 1.133 | 1.161 |
| tZOGR | 0.177 | 0.177 | 0.159 | 0.313 | 0.322 | 0.342 | 0.234 | 0.258 | 0.225 | 0.356 | 0.369 | 0.332 | 0.420 | 0.415 | 0.369 |
| tZ9G | 0.866 | 0.547 | 0.793 | 0.810 | 0.808 | 0.779 | 1.144 | 1.261 | 1.098 | 1.382 | 1.371 | 1.625 | 1.631 | 1.540 | 1.803 |
| DHZ | 0.187 | 0.178 | 0.142 | 0.911 | 1.001 | 0.867 | 1.133 | 1.147 | 1.128 | 1.722 | 1.640 | 1.669 | 2.032 | 1.843 | 1.853 |
| DHZR | 0.203 | 0.177 | 0.182 | 0.230 | 0.200 | 0.254 | 0.267 | 0.295 | 0.257 | 0.444 | 0.390 | 0.380 | 0.524 | 0.439 | 0.422 |
| DHZOG | 0.067 | 0.064 | 0.050 | 0.442 | 0.466 | 0.422 | 0.089 | 0.098 | 0.085 | 0.135 | 0.140 | 0.126 | 0.160 | 0.158 | 0.140 |
| DHZOGR | 1.051 | 1.710 | 1.566 | 1.450 | 1.336 | 1.323 | 1.387 | 1.530 | 1.332 | 2.108 | 2.188 | 1.971 | 2.023 | 2.459 | 2.187 |
| DHZ7G | 0.376 | 0.457 | 0.398 | 0.542 | 0.514 | 0.611 | 0.496 | 0.547 | 0.476 | 0.754 | 0.782 | 0.705 | 0.890 | 0.879 | 0.782 |
| DHZ9G | 0.348 | 0.426 | 0.367 | 0.456 | 0.479 | 0.445 | 0.460 | 0.507 | 0.441 | 0.699 | 0.725 | 0.653 | 0.825 | 0.815 | 0.725 |
| IP | 0.534 | 0.909 | 0.987 | 0.992 | 0.773 | 0.882 | 0.705 | 0.777 | 0.677 | 1.071 | 1.112 | 1.001 | 1.264 | 1.249 | 1.468 |
| IPR7G | 0.272 | 0.145 | 0.197 | 0.159 | 0.164 | 0.167 | 0.359 | 0.396 | 0.345 | 0.458 | 0.443 | 0.510 | 0.540 | 0.498 | 0.455 |
| oT | 0.361 | 0.307 | 0.344 | 0.338 | 0.346 | 0.337 | 0.477 | 0.526 | 0.458 | 0.725 | 0.752 | 0.677 | 0.855 | 0.845 | 0.752 |
| mT | 0.266 | 0.261 | 0.238 | 0.264 | 0.293 | 0.250 | 0.351 | 0.387 | 0.337 | 0.534 | 0.554 | 0.499 | 0.552 | 0.460 | 0.554 |
| pT | 0.183 | 0.317 | 0.211 | 0.171 | 0.178 | 0.178 | 0.241 | 0.266 | 0.232 | 0.367 | 0.380 | 0.343 | 0.326 | 0.428 | 0.380 |
| BA | 0.829 | 0.889 | 0.663 | 0.775 | 0.790 | 0.560 | 1.094 | 1.207 | 1.051 | 1.663 | 1.726 | 1.555 | 1.963 | 1.940 | 1.726 |
| GA3 | 6.032 | 7.124 | 7.527 | 5.640 | 7.021 | 6.361 | 9.962 | 8.782 | 7.644 | 11.143 | 12.559 | 11.313 | 13.148 | 14.116 | 12.557 |
| SA | 2.305 | 1.273 | 1.664 | 1.462 | 1.433 | 1.510 | 2.485 | 2.741 | 2.386 | 3.777 | 3.920 | 3.531 | 4.568 | 5.893 | 4.079 |
