# Supplementary material for: Hormone variation in Robinia pseudoacacia L. (Fabaceae) leaves during gall formation by Obolodiplosis robiniae (Haldeman) (Diptera: Cecidomyiidae)
Source: Sci Rep. 2026 Mar 12;16:8815. doi: 10.1038/s41598-026-38156-9 (PMC12982751; doi:10.1038/s41598-026-38156-9)
Supplement: Supplementary file 2 — Supplementary Material 2 [file 41598_2026_38156_MOESM2_ESM.pdf]

# = Shimadzu LabSolutions Quant. Browser Data Report =

Acquired by	: System Administrator
Data Acquired	: 6/28/2022 10:53:33 PM
Sample Type	: Unknown
Sample Name	: GAL
Sample ID	: 003
Sample Amount	: 1
Dilution Factor	: 1
Vial#	: 6
Injection Volume	: 10 uL
Data Filename	: Phytohormones in GALL Robinia.lcd
Method Filename	: System Administrator
Processed by	: 7/28/2022 3:49:29 PM
Modified Date	

31.0000>440.1000(+)@28

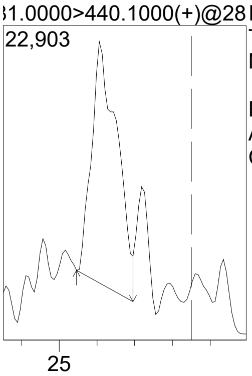

ID#:1 m/z: 481.0000>440.1000
Type:Target
Name:BL

Ret.Time:26.000
Area:1235432
Conc.:2.353 ppb

)5.1000>459.1500(+)@25

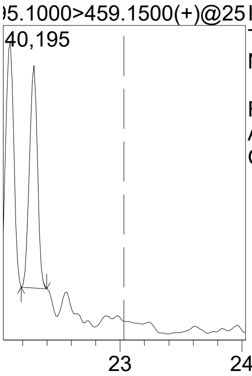

ID#:2 m/z: 495.1000>459.1500
Type:Target
Name:HBL

Ret.Time:22.000
Area:34322
Conc.:3.312 ppb

0000>147.9500(+)@7 (1)

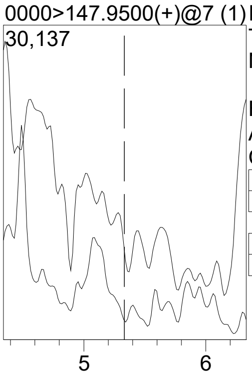

ID#:3 m/z: 222.0000>136.0500
Type:Target
Name:DHZ

Ret.Time:5.205
Area:218314
Conc.:2.301ppb

#	m/z	Area
1	222.00>147.95	5975

‘ª’è%
17.52

0000>220.1000(+)@2 (1)

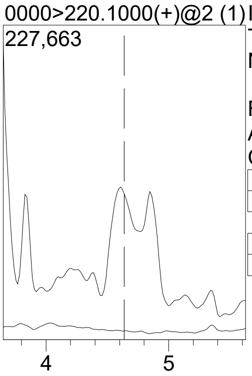

ID#:4 m/z: 382.0000>136.1000
Type:Target
Name:tzGLU

Ret.Time:6.187
Area:2343150
Conc.:5.695ppb

#	m/z	Area
1	382.00>220.10	356442

‘ª’è%
65.85

31.1000>445.2000(+)@24

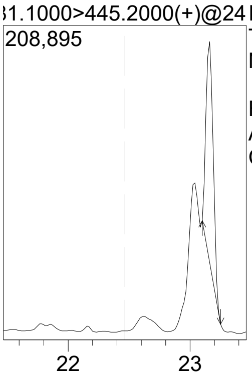

ID#:5 m/z: 481.1000>445.2000
Type:Target
Name:epiBL

Ret.Time:23.000
Area:4232
Conc.:4.032 ppb

35.1000>429.3500(+)@26

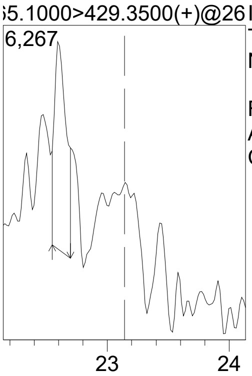

ID#:6 m/z: 465.1000>429.3500
Type:Target
Name:epiCS

Ret.Time:23.200
Area:54344
Conc.:1.532 ppb

.0000>118.9000(+)@8 (1) ID#:7 m/z: 220.0000>136.1500

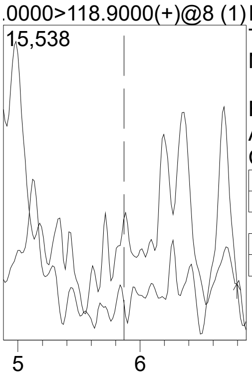

Type:Target
Name:TransZEA

Ret.Time:7.089
Area:3779034
Conc.:0.734ppb

#	m/z	Area
1	220.00>118.90	118315

‘ª’è%
23.99

9000>76.9500(+)@19 (1) ID#:8 m/z: 175.9000>130.3000

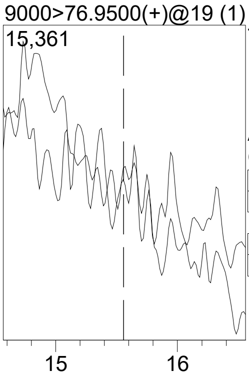

Type:Target
Name:IAA

Ret.Time:15.467
Area:228692
Conc.:6.472ppb

#	m/z	Area
1	175.90>76.95	10363

‘ª’è%
36.62

9000>77.0500(+)@20 (1) ID#:9 m/z: 189.9000>130.2500

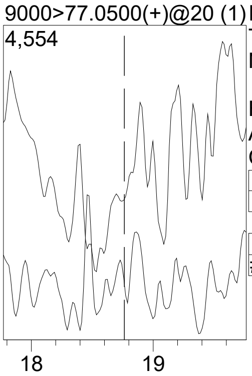

Type:Target
Name:IPA

Ret.Time:19.089
Area:12004
Conc.:1.373ppb

#	m/z	Area
1	189.90>77.05	109725

‘ª’è%
#######

1000>143.2000(-)@34 (1) ID#:10 m/z: 345.1000>239.0000

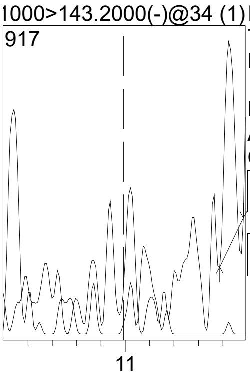

Type:Target
Name:GA

Ret.Time:11.000
Area:34221
Conc.:1.321 ppb

#	m/z	Area
1	345.10>143.20	30

‘ª’è%
4.67

137.0000>92.9500(-)@37 ID#:11 m/z: 137.0000>92.9500

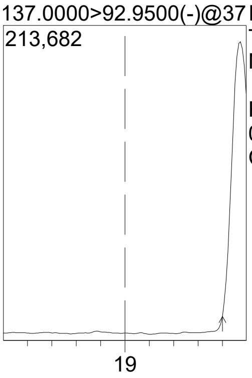

Type:Target
Name:SA

Ret.Time:19.840
0 Area:4543333
Conc.:4.672 ppb

9000>43.0000(+)@12 (1) ID#:12 m/z: 351.9000>135.9500

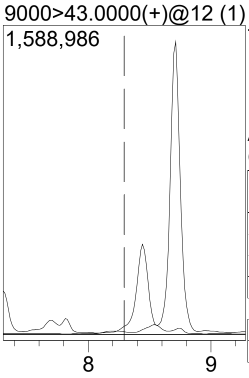

Type:Target
Name:tZR

Ret.Time:8.701
Area:48070519
Conc.:38.662ng/ml

#	m/z	Area
1	351.90>220.00	8740291
2	351.90>118.90	0
3	351.90>202.25	0
4	351.90>148.10	0
5	351.90>43.00	0

‘ª’è%
111.64

1.9000>43.2000(+)@3 (1) ID#:13 m/z: 381.9000>136.0000

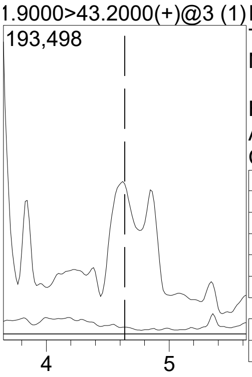

Type:Target
Name:tZ9G

Ret.Time:4.578
Area:2934418
Conc.:10.031ng/ml

#	m/z	Area
1	381.90>220.00	347481
2	381.90>119.05	0
3	381.90>202.00	0
4	381.90>148.00	0
5	381.90>43.20	0

‘ª’è%
96.66

1.9000>43.2000(+)@4 (1) ID#:14 m/z: 381.9000>220.2000

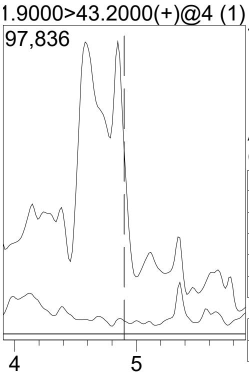

Type:Target
Name:cZ9G

Ret.Time:4.829
Area:1356524
Conc.:10.917ng/ml

#	m/z	Area
1	381.90>135.95	96140
2	381.90>118.90	0
3	381.90>202.10	0
4	381.90>185.05	0
5	381.90>43.20	0

‘ª’è%
71.26

000>185.2000(+)@15 (1) ID#:15 m/z: 351.9000>220.0500

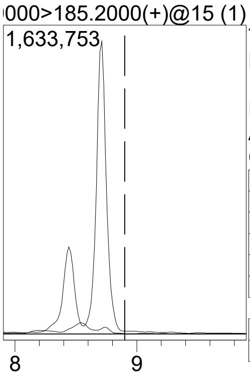

Type:Target
Name:cZR

Ret.Time:8.822
Area:1415584
Conc.:0.683ng/ml

#	m/z	Area
1	351.90>135.95	182590
2	351.90>119.00	0
3	351.90>202.05	0
4	351.90>43.00	0
5	351.90>185.20	0

‘ª’è%
68.70

000>137.2000(+)@13 (1) ID#:16 m/z: 353.9000>222.0000

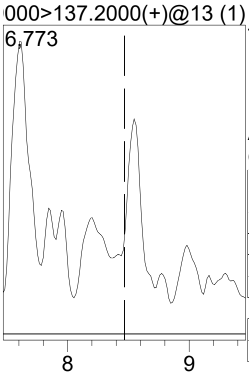

Type:Target
Name:DHZR

Ret.Time:8.332
Area:408542
Conc.:2.503ng/ml

#	m/z	Area
1	353.90>135.95	0
2	353.90>148.05	0
3	353.90>69.10	0
4	353.90>119.05	0
5	353.90>137.20	0

‘ª’è%
0.00

3.9000>85.0000(+)@9 (1) ID#:17 m/z: 383.9000>222.1000

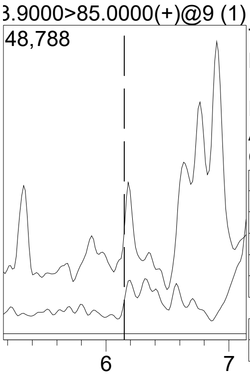

Type:Target
Name:DHZOG

Ret.Time:6.82
Area: 7682623
Conc.:2.758ng/ml

#	m/z	Area
1	383.90>136.00	55082
2	383.90>148.00	0
3	383.90>69.10	0
4	383.90>204.20	0
5	383.90>85.00	0

‘ª’è%
#######

.9000>118.8500(+)@5 (1) ID#:18 m/z: 383.9000>222.1000

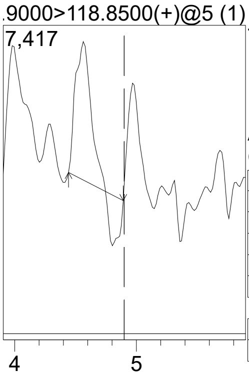

Type:Target
Name:DHZG9

Ret.Time:4.900
Area:1522633
Conc.:2.6323ng/ml

#	m/z	Area
1	383.90>136.00	0
2	383.90>148.10	0
3	383.90>137.05	0
4	383.90>68.95	0
5	383.90>118.85	0

‘ª’è%
0.00

.9000>119.0000(+)@6 (1) ID#:19 m/z: 383.9000>222.2000

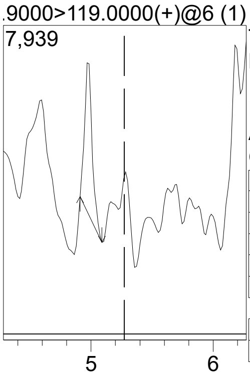

Type:Target
Name:DHZ7G

Ret.Time:4.9210
Area:32672
Conc.:0.42312 ng/ml

#	m/z	Area
1	383.90>136.00	0
2	383.90>148.05	0
3	383.90>137.20	0
4	383.90>69.10	0
5	383.90>119.00	0

‘ª’è%
0.00

9000>39.0500(+)@16 (1) ID#:20 m/z: 203.9000>135.9500

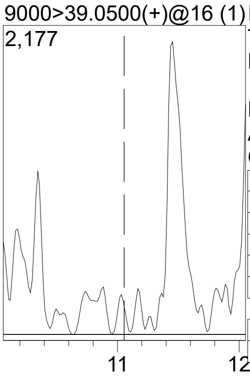

Type:Target
Name:IP

Ret.Time:11.372
Area:277447
Conc.:0.751ng/ml

#	m/z	Area
1	203.90>119.05	0
2	203.90>69.10	0
3	203.90>148.15	0
4	203.90>41.00	0
5	203.90>39.05	0

‘ª’è%
0.00

9000>226.9000(+)@1 (1) ID#:21 m/z: 335.9000>136.0000

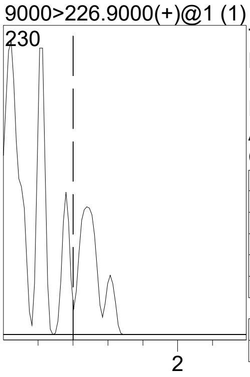

Type:Target
Name:IPR

Ret.Time:1.320
Area:142402
Conc.:0.32ng/ml

#	m/z	Area
1	335.90>204.05	0
2	335.90>148.20	0
3	335.90>119.00	0
4	335.90>69.05	0
5	335.90>226.90	0

‘ª’è%
0.00

.8000>41.0000(+)@11 (1) ID#:22 m/z: 365.8000>136.0500

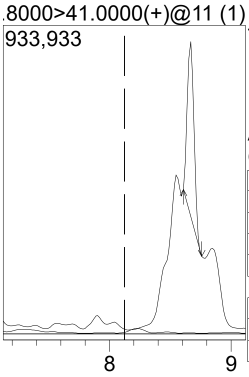

Type:Target
Name:IPR7G

Ret.Time:8.7120
Area:6464
Conc.:0.32ng/ml

#	m/z	Area
1	365.80>204.00	824
2	365.80>119.00	0
3	365.80>69.05	0
4	365.80>41.00	0

‘ª’è%
0.14
0.00

9000>92.0500(+)@10 (1) ID#:23 m/z: 241.9000>136.0000

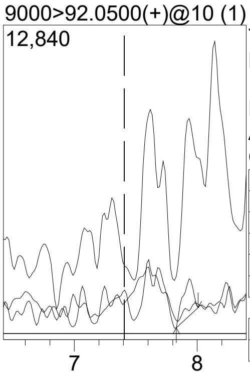

Type:Target
Name:OT

Ret.Time:8.140
Area:4562
Conc.:1.25ng/ml

#	m/z	Area
1	241.90>107.00	37
2	241.90>119.00	0
3	241.90>77.05	3761
4	241.90>79.10	0
5	241.90>92.05	0

‘ª’è%
2.09

000>119.0500(+)@14 (1) ID#:24 m/z: 241.8000>107.0000

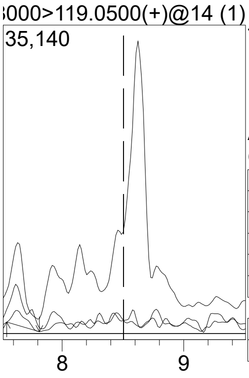

Type:Target
Name:MT

Ret.Time:8.500
Area:563221
Conc.:2.06ng/ml

#	m/z	Area
1	241.80>77.05	6137
2	241.80>136.05	3544
3	241.80>79.15	0
4	241.80>51.10	0
5	241.80>119.05	0

‘ª’è%
380.71

9000>92.0500(+)@17 (1) ID#:25 m/z: 241.9000>107.0000

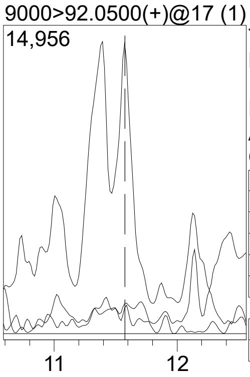

Type:Target
Name:PT

Ret.Time:11.234
Area:59118
Conc.:1.564ng/ml

#	m/z	Area
1	241.90>135.95	27189
2	241.90>77.05	21550
3	241.90>119.00	0
4	241.90>79.10	0
5	241.90>92.05	0

‘ª’è%
179.06

000>148.1000(+)@18 (1) ID#:26 m/z: 225.9000>90.9500

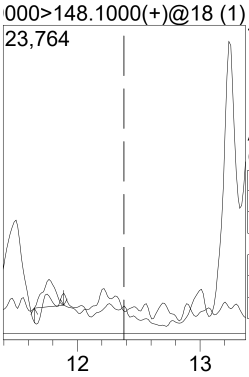

Type:Target
Name:BA

Ret.Time:13.120
Area:57543
Conc.:1.78ng/ml

#	m/z	Area
1	225.90>64.95	781
2	225.90>148.10	0

‘ª’è%
35.61
0.00

000>103.0500(+)@21 (1) ID#:27 m/z: 203.8000>186.0000

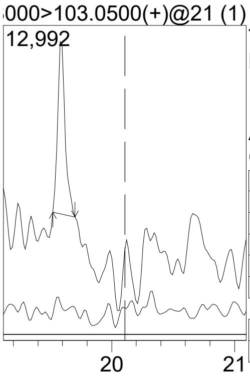

Type:Target
Name:IBA

Ret.Time:19.870
Area:56432345
Conc.:6.312ng/ml

#	m/z	Area
1	203.80>130.00	503
2	203.80>144.10	0
3	203.80>117.00	0
4	203.80>168.00	0
5	203.80>103.05	0

‘ª’è%
6.62

000>313.1000(+)@22 (1) ID#:28 m/z: 467.1000>431.1000

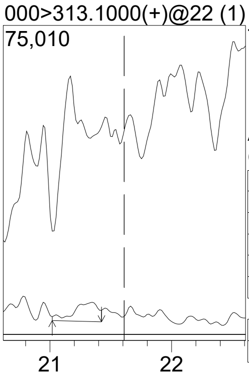

Type:Target
Name:NorBL

Ret.Time:21.320
Area:6935432
Conc.:3.21ng/ml

#	m/z	Area
1	467.10>81.20	2782
2	467.10>413.15	0
3	467.10>315.10	0
4	467.10>95.15	0
5	467.10>313.10	0

‘ª’è%
113.64

000>388.9000(+)@27 (1) ID#:29 m/z: 434.7000>394.0500

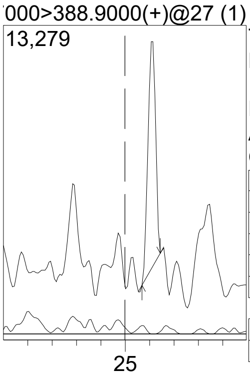

Type:Target
Name:6dTY

Ret.Time:25.275
Area:117932
Conc.:0.16ng/ml

#	m/z	Area
1	434.70>352.75	0
2	434.70>312.25	0
3	434.70>373.00	0
4	434.70>416.50	0
5	434.70>388.90	0

‘ª’è%
0.00

000>309.1000(+)@23 (1) ID#:30 m/z: 432.7000>365.1500

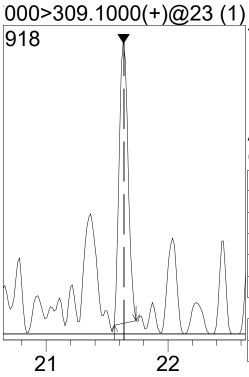

Type:Target
Name:CT

Ret.Time:21.634
Area:4233
Conc.:4.194ng/ml

#	m/z	Area
1	432.70>391.85	0
2	432.70>351.00	0
3	432.70>368.60	0
4	432.70>415.05	0
5	432.70>309.10	0

‘ª’è%
0.00

9000>133.9500(-)@29 (1) ID#:31 m/z: 541.9000>200.1500

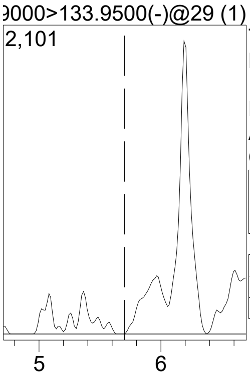

Type:Target
Name:tZ9GOG

Ret.Time:6.100
Area:3322323
Conc.:2.04ng/ml

#	m/z	Area
1	541.90>380.10	0
2	541.90>133.95	0

‘a’è%
0.00
0.00

1000>134.1000(-)@30 (1) ID#:32 m/z: 512.1000>380.1000

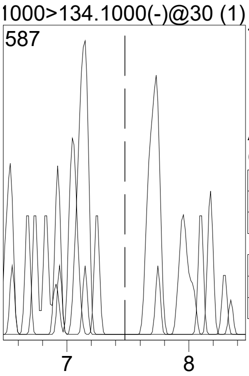

Type:Target
Name:tZOGR

Ret.Time:7.707
Area:19751
Conc.:4.940ng/ml

#	m/z	Area
1	512.10>200.05	2290
2	512.10>134.10	347

‘a’è%
88.08
13.35

1000>117.0500(-)@33 (1) ID#:33 m/z: 218.1000>133.0500

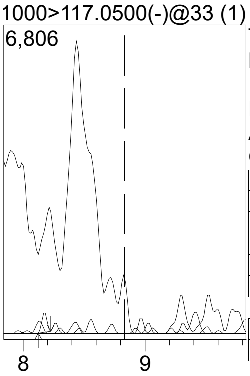

Type:Target
Name:cZ

Ret.Time:8.510
Area:153112
Conc.:5.68ng/ml)

#	m/z	Area
1	218.10>200.00	141
2	218.10>134.05	0
3	218.10>132.00	0
4	218.10>188.00	0
5	218.10>117.05	0

‘a’è%
29.38

1000>218.1500(-)@32 (1) ID#:34 m/z: 512.1000>380.1000

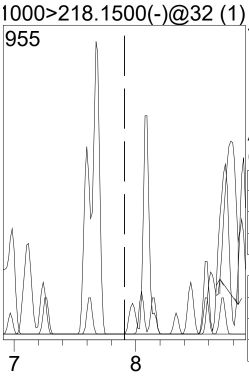

Type:Target
Name:cZOGR

Ret.Time:8.230
Area:892311
Conc.:0.32ng/ml

#	m/z	Area
1	512.10>200.05	0
2	512.10>134.15	0
3	512.10>218.15	0

‘a’è%
0.00
0.00
0.00

.1000>89.0500(-)@31 (1) ID#:35 m/z: 514.1000>382.0500

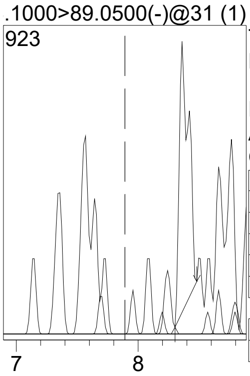

Type:Target
Name:DHZOGR

Ret.Time:8.467
Area:1231
Conc.:0.32ng/ml

#	m/z	Area
1	514.10>202.00	0
2	514.10>220.00	0
3	514.10>134.00	0
4	514.10>59.00	0
5	514.10>89.05	0

‘a’è%
0.00

.2000>89.1000(-)@35 (1) ID#:36 m/z: 135.2000>91.0000

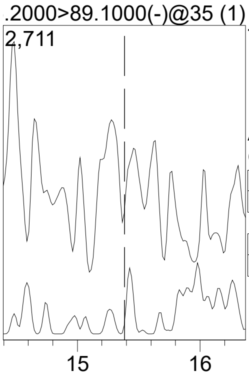

Type:Target
Name:PAA

Ret.Time:15.346
Area:40251
Conc.:41.807ng/ml

#	m/z	Area
1	135.20>89.10	408

‘a’è%
12.11

2000>188.1500(-)@36 (1) ID#:37 m/z: 263.2000>153.0500

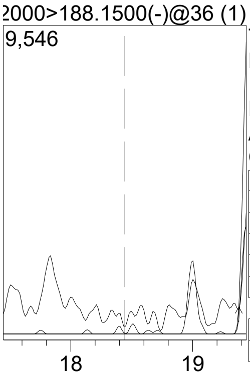

Type:Target
Name:ABA

Ret.Time:19.010
Area:370421
Conc.:0.25ng/ml

#	m/z	Area
1	263.20>219.05	660
2	263.20>204.15	1287
3	263.20>203.00	0
4	263.20>201.10	0
5	263.20>188.15	0

‘a’è%
21.80
